# Distinct phenotypic signatures between anejaculation and premature ejaculation: evidence from a large clinical cohort

**DOI:** 10.3389/frph.2025.1711365

**Published:** 2025-10-29

**Authors:** Tianbiao Zhang, Ziang Shi, Rui Wang, Tao Zheng, Yonghao Nan, Kunlong Lv

**Affiliations:** Department of Andrology, The First Affiliated Hospital of Zhengzhou University, Zhengzhou, China

**Keywords:** anejaculation, premature ejaculation, vibration perception threshold, spinal MRI, depression

## Abstract

**Background:**

Anejaculation (AE) and premature ejaculation (PE) are clinically distinct but mechanistically complex disorders. While both contribute substantially to male sexual health burden, their comparative profiles have not been systematically delineated in large cohorts.

**Methods:**

We retrospectively analyzed 542 men (AE = 249, PE = 293) at a tertiary andrology clinic. All participants underwent vibration perception threshold (VPT) testing at ten standardized sites, spinal MRI reviewed by blinded radiologists, expressed prostatic secretion microscopy with supportive ultrasound for prostatitis, and validated psychological assessments (PHQ-9, GAD-7, SDI-2). Statistical comparisons used Mann–Whitney *U* tests, chi-squared tests, and multivariate analysis of variance (MANOVA).

**Results:**

AE patients exhibited higher composite VPT thresholds than PE (7.12 ± 1.75 vs. 6.60 ± 1.26, *p* < 0.001), with MANOVA confirming distinct sensory profiles (Wilks’ *λ* = 0.907, *p* < 0.001). Cervical-only abnormalities were markedly more frequent in PE (33.4% vs. 2.8%), whereas AE more often showed either no abnormality (45.8% vs. 28.0%) or combined cervical–lumbar involvement (14.5% vs. 7.5%; overall *χ*^2^ = 84.46, *p* < 0.001). Chronic prostatitis was present in nearly half of AE cases but only one fifth of PE (47.4% vs. 20.1%, *p* < 0.001). Depressive symptoms were modestly higher in AE (PHQ-9: 8.31 ± 5.96 vs. 7.15 ± 5.65, *p* = 0.024), while sexual desire and anxiety scores did not differ significantly.

**Conclusions:**

AE and PE display distinct clinical signatures. AE was linked to higher vibration thresholds, greater prevalence of prostatitis, and elevated depressive symptoms, while PE was predominantly associated with isolated cervical spine abnormalities. Recognizing these patterns may refine clinical assessment and guide more individualized management.

## 1 Introduction

Ejaculation is a complex physiological event that integrates peripheral genital sensation, spinal pattern generators, and supraspinal regulatory networks. Disruption of this finely coordinated process can manifest as a variety of ejaculatory disorders, including premature ejaculation (PE), delayed ejaculation (DE), anejaculation (AE), and retrograde ejaculation (RE). These conditions differ markedly in prevalence, symptom burden, and clinical consequences. PE is the most common male sexual dysfunction worldwide, affecting up to 20%–30% of men and often leading to significant personal distress and relational strain (1). AE, although relatively rare, is highly debilitating, defined as the persistent or recurrent inability to ejaculate despite sufficient stimulation, and is strongly linked to infertility and impaired quality of life (2). Unlike the diagnostic challenges sometimes faced with erectile dysfunction, PE and AE are usually distinguishable in routine clinical settings by their symptom profiles. What remains uncertain—and clinically important—is the extent to which their underlying pathophysiological mechanisms diverge.

Understanding such divergence requires attention to multiple domains. At the peripheral level, genital sensory input is critical for initiating ejaculatory reflexes. Vibration perception threshold (VPT) testing, widely used in clinical neuro-urology, offers an objective measure of peripheral sensory function and has been explored as a potential adjunct in the evaluation of men with ejaculatory dysfunction (3). The assumption has been that VPT might help discriminate between disorders with peripheral sensory involvement and those driven primarily by central or psychological mechanisms (4). However, evidence supporting this role is limited and inconsistent. Observational studies suggest that VPT values in men with AE and PE often overlap considerably, casting doubt on its clinical discriminative power (5).

Beyond peripheral sensory function, other mechanisms merit consideration. Spinal abnormalities, such as cervical or lumbar degenerative changes, may alter reflex integration; chronic inflammatory conditions like prostatitis have been implicated in ejaculatory complaints; and psychological factors, including depression, anxiety, and sexual desire, frequently intersect with sexual functioning. Yet, despite these plausible contributors, the literature remains fragmented, hampered by small sample sizes, heterogeneous methods, and limited cross-domain integration (6).

In this context, a comprehensive evaluation that examines AE and PE across neurological, structural, inflammatory, and psychological dimensions is urgently needed. Such an approach, rather than redefining diagnosis, seeks to clarify mechanistic contrasts between these two clinically distinct but pathophysiologically complex disorders. By systematically addressing these domains within a large patient cohort, the present study aims to refine understanding of AE and PE and to inform more precise clinical management.

## 2 Materials and methods

### 2.1 Study design and participants

This was a single-center, retrospective, cross-sectional study conducted in the Department of Andrology, First Affiliated Hospital of Zhengzhou University between January 2025 and June 2025. Consecutive men presenting to the specialty clinic and clinically diagnosed with anejaculation (AE) or premature ejaculation (PE) were included. AE was defined as the persistent or recurrent inability to ejaculate despite adequate sexual stimulation with associated distress or functional impairment (7). PE was defined according to the International Society for Sexual Medicine (ISSM) criteria, covering lifelong and acquired forms with clinically relevant rapid ejaculation and accompanying distress or interpersonal difficulty (6). Exclusion criteria were pre-specified: neurological diseases known to affect sensory or motor pathways (e.g., multiple sclerosis, spinal cord injury), prior pelvic or spinal surgery or major congenital genitourinary malformations, and severe psychiatric illness. Use of medications with established effects on ejaculation (e.g., selective serotonin reuptake inhibitors, tricyclic antidepressants, *α*-adrenergic blockers) led to exclusion if taken during the assessment window. All data were extracted from routine-care records and de-identified prior to analysis. This non-interventional, retrospective analysis adhered to institutional and national regulations and to the principles of the Declaration of Helsinki; no research procedures were performed outside standard clinical care.

### 2.2 Clinical assessments

Vibration perception threshold (VPT) was obtained with a standardized vibrotactile analyzer (Bio-Thesiometer, Bio-Medical Instrument Co., Newbury, OH, USA) in a quiet, temperature-controlled room after brief acclimatization. Each participant was assessed at ten predefined sites: the glans penis at 12, 3, 6, and 9 o'clock positions; the penile root at 12, 3, 6, and 9 o'clock positions; and the left and right index fingertips as reference sites. The instrument delivered a fixed-frequency sinusoidal stimulus; amplitude was raised in small steps following a method-of-limits staircase. At each site, three trials were administered with short inter-trial intervals, and the site-specific threshold was defined as the lowest amplitude perceived in at least two of three trials. The probe was applied perpendicular to the skin with light, uniform pressure throughout testing, and basic functional checks recommended by the manufacturer were completed at the start of clinic sessions.

Spinal MRI was acquired on a 3.0-Tesla clinical system (SIGNA™ Premier, GE Healthcare) using standardized spine protocols, including sagittal T1- and T2-weighted sequences, a sagittal fat-suppressed/STIR sequence, and axial T2-weighted images targeted to levels with suspected pathology. Typical sagittal parameters were 3–4 mm slice thickness with ≤0.5 mm interslice gap; axial sections were aligned to the involved discs/foramina. Coverage included cervical levels C3–C8 and the thoracolumbar junction T12–L2. Two senior radiologists, blinded to AE/PE grouping, reviewed all scans independently and resolved discrepancies by consensus. Findings were summarized into four mutually exclusive categories: cervical abnormality only, lumbar abnormality only, both cervical and lumbar abnormalities, or no abnormality. Abnormalities comprised intervertebral disc protrusion or bulging, spinal canal or foraminal stenosis, and nerve root compression (8).

Chronic prostatitis status was abstracted from routine andrology records. Expressed prostatic secretions (EPS) were obtained after standard prostatic massage and examined microscopically at ×400 magnification; a count of ≥10 leukocytes per high-power field was used as the diagnostic threshold. Sonographic features typical of chronic prostatitis—heterogeneous echotexture, increased vascularity, or intraprostatic calcifications—were recorded when present to support the clinical designation (9). No additional laboratory procedures beyond routine care were performed for this study.

Psychological status was assessed using validated Chinese-language versions of the Patient Health Questionnaire-9 (PHQ-9) for depressive symptoms, the Generalized Anxiety Disorder-7 (GAD-7) for anxiety symptoms, and the Sexual Desire Inventory-2 (SDI-2) for sexual desire (10). Trained staff administered all instruments according to standard instructions.

### 2.3 Statistical analysis

Continuous variables, including VPT values at each anatomical site, the composite mean VPT, and psychological scale scores (PHQ-9, GAD-7, SDI-2), were compared between AE and PE groups using Mann–Whitney U tests. For VPT, site-specific results were analyzed individually, and a ten-site average was calculated to provide a summary measure. In addition, overall differences in the sensory profile were evaluated using multivariate analysis of variance (MANOVA) with all ten VPT variables entered as the dependent vector. MRI findings, categorized into four mutually exclusive groups, and chronic prostatitis status were compared using chi-squared tests. All statistical tests were two-sided, and a *p*-value < 0.05 was considered statistically significant. Exact *p* values are reported unless smaller than 0.001, in which case they are presented as *p* < 0.001. Analyses were conducted using R software (version 4.3.0; R Foundation for Statistical Computing, Vienna, Austria).

## 3 Results

### 3.1 Vibration perception threshold (VPT)

VPT results at ten anatomical sites are summarized in Table 1. After FDR correction, several penile sites demonstrated significantly higher thresholds in the AE group compared with PE. Specifically, differences were observed at the glans 12 o'clock (7.80 ± 3.13 vs. 7.15 ± 2.99, *p* = 0.004, *r* = −0.163), glans 6 o'clock (9.23 ± 5.47 vs. 7.81 ± 2.51, *p* = 0.004, *r* = −0.156), glans 9 o'clock (8.64 ± 5.22 vs. 8.44 ± 6.44, *p* = 0.032, *r* = −0.116), root 12 o'clock (6.16 ± 2.07 vs. 5.79 ± 2.36, *p* = 0.018, *r* = −0.130), root 3 o'clock (6.85 ± 3.74 vs. 6.07 ± 2.69, *p* = 0.004, *r* = −0.159), and root 6 o'clock (8.33 ± 6.20 vs. 6.68 ± 3.17, *p* = 0.004, *r* = −0.169). In contrast, thresholds at glans 3 o'clock (*p* = 0.079), root 9 o'clock (*p* = 0.529), and both index fingers (*p* > 0.5) did not differ significantly.

**Table 1:** Vibration perception threshold (VPT) values at ten anatomical sites in AE and PE patients.

VPT site	AE (mean ± SD)	PE (mean ± SD)	*p* (FDR)
Glans 12 o'clock	7.80 ± 3.13	7.15 ± 2.99	0.004
Glans 3 o'clock	7.79 ± 3.55	7.20 ± 2.54	0.079
Glans 6 o'clock	9.23 ± 5.47	7.81 ± 2.51	0.004
Glans 9 o'clock	8.64 ± 5.22	8.44 ± 6.44	0.032
Root 12 o'clock	6.16 ± 2.07	5.79 ± 2.36	0.018
Root 3 o'clock	6.85 ± 3.74	6.07 ± 2.69	0.004
Root 6 o'clock	8.33 ± 6.20	6.68 ± 3.17	0.004
Root 9 o'clock	6.14 ± 3.19	6.15 ± 2.94	0.529
Left index finger	5.41 ± 1.64	5.38 ± 1.96	0.534
Right index finger	4.86 ± 1.12	5.29 ± 1.99	0.529
Average VPT	7.12 ± 1.75	6.60 ± 1.26	<0.001

Values are presented as mean ± SD. Between-group comparisons were performed with Mann–Whitney *U* tests. Composite mean values represent the average across all ten test sites. *p*-values <0.05 were considered statistically significant after FDR correction.

When all ten test sites were averaged, AE patients exhibited higher overall thresholds than PE patients (7.12 ± 1.75 vs. 6.60 ± 1.26, *p* < 0.001, *r* = −0.243). MANOVA confirmed an overall difference in the sensory profile between groups [Wilks' *λ* = 0.907, F(10, 531) = 5.444, *p* < 0.001; Pillai's trace consistent]. These findings indicate that although not every individual site distinguished between conditions, the composite sensory profile differed significantly between AE and PE.

### 3.2 Spinal MRI

MRI findings showed strikingly different patterns between the two groups (*χ*^2^ = 84.46, df = 3, *p* < 0.001; Table 2). In AE, nearly half of the patients (45.8%) had no detectable abnormality, whereas this was seen in only 28.0% of PE. Conversely, isolated cervical lesions were uncommon in AE (2.8%) but present in one third of PE patients (33.4%, *p* < 0.001), constituting the most marked disparity. Dual involvement of both cervical and lumbar segments was more frequent in AE (14.5% vs. 7.5%, *p* = 0.009). Isolated lumbar changes occurred at similar rates (36.9% vs. 31.1%, *p* = 0.149).

**Table 2:** Distribution of spinal MRI findings in AE and PE patients.

MRI category	AE *n*	AE %	PE *n*	PE %
No abnormality	114	45.8	82	28
Lumbar only	92	36.9	91	31.1
Cervical only	7	2.8	98	33.4
Cervical + Lumbar	36	14.5	22	7.5

Findings were categorized into four mutually exclusive groups: no abnormality, cervical abnormality only, lumbar abnormality only, and combined cervical–lumbar abnormalities. Comparisons between groups were conducted with chi-squared tests.

These distributions suggest that AE is characterized by either structurally normal imaging or combined cervical–lumbar involvement, whereas PE is most strongly associated with cervical pathology alone.

### 3.3 Chronic prostatitis

The prevalence of chronic prostatitis differed sharply between groups (Table 3). Nearly half of AE patients were positive on expressed prostatic secretion microscopy and ultrasound criteria (118/249, 47.4%), compared with only one fifth of PE patients (59/293, 20.1%; *χ*^2^ = 45.46, *p* < 0.001). Conversely, prostatitis was absent in four out of five PE patients but in only half of AE cases, underscoring a significant difference in prevalence between the groups.

**Table 3:** Prevalence of chronic prostatitis in AE and PE patients.

Chronic prostatitis	AE n	AE %	PE n	PE %
Positive	118	47.4	59	20.1
Negative	131	52.6	234	79.9

Diagnosis was based on expressed prostatic secretion microscopy (≥10 leukocytes per high-power field) supported by characteristic ultrasound features. Group differences were assessed with chi-squared tests.

### 3.4 Psychological scales

Group comparisons of psychological measures are summarized in Table 4. AE patients reported significantly higher depressive symptoms than PE (PHQ-9: 8.31 ± 5.96 vs. 7.15 ± 5.65, *p* = 0.024, *r* = −0.108). However, levels of sexual desire (SDI-2: 36.64 ± 7.14 vs. 37.03 ± 6.58, *p* = 0.633) and anxiety (GAD-7: 5.56 ± 4.81 vs. 5.25 ± 4.78, *p* = 0.440) were broadly similar across groups.

**Table 4:** Psychological scale scores in AE and PE patients.

Scale	AE (mean ± SD)	PE (mean ± SD)	*p* value
SDI-2 (Sexual Desire Score)	36.64 ± 7.14	37.03 ± 6.58	0.633
PHQ-9 (Depression Score)	8.31 ± 5.96	7.15 ± 5.65	0.024
GAD-7 (Anxiety Score)	5.56 ± 4.81	5.25 ± 4.78	0.440

Scores are presented as mean ± SD. Group comparisons were conducted with Mann–Whitney *U* tests; effect size (r) is shown where relevant.

Taken together, these findings suggest that depressive symptom burden may play a role in differentiating AE from PE, while sexual desire and anxiety appear to be comparable across conditions. Importantly, the inclusion of multiple validated scales ensured that psychological evaluation was comprehensive, capturing both mood- and drive-related domains rather than relying on a single dimension.

## 4 Discussion

Ejaculation is organized by a spinal network that integrates genital afference with descending supraspinal control and coordinates sympathetic, parasympathetic, and somatic outflow for emission and expulsion. In humans and animal models, a “spinal ejaculation generator” in the lumbosacral cord receives input from the dorsal penile nerve and communicates with supraspinal nodes (e.g., medial preoptic area, paraventricular hypothalamus), providing a biologically coherent framework in which differences in sensory inflow could map onto ejaculatory phenotypes without implying simple causality (11). Within this framework, the vibration-based sensory signal recorded by VPT represents a quantitative readout of this afferent limb, linking peripheral mechanoreceptor activity to the spinal generator's excitability and thereby offering a clinically accessible index of sensory drive strength.

Against that backdrop, our data show a measurable separation between AE and PE when VPT is considered as an aggregate signal (ten-site mean and a multivariate profile), while single-site discrimination is heterogeneous. This pattern is congruent with the idea that vibration detection—largely a large-fiber (Aβ) mechanosensory readout—samples only one slice of the afferent landscape and is modulated by central gating (12); it should not be over-interpreted as a direct test of penile nerve conduction. Classic comparative work already cautioned that penile biothesiometry cannot replace neuro-urophysiological studies such as pudendal somatosensory evoked potentials or sacral reflex latency testing (13); our findings are consistent with treating VPT as a phenotypic quantifier rather than a surrogate neurophysiological assay. In AE, elevated composite thresholds indicate attenuated input from dorsal-penile Aβ fibers to the S2–S4 spinal segments, potentially reducing emission-triggering probability; in PE, preserved or lower thresholds relative to AE may instead reflect enhanced supraspinal facilitation that prematurely activates the same spinal circuitry. Thus, VPT profiles delineate opposite ends of a sensory-gain spectrum that maps directly onto the ejaculatory phenotype.

A practical implication is methodological: if VPT is to add durable value, it should be analyzed the way quantitative sensory testing (QST) is analyzed elsewhere in neurosensory medicine—standardized acquisition, explicit control of multiplicity across sites, and pattern-level interpretation (e.g., composite indices or multivariate profiles) instead of privileging any single point. The DFNS QST framework provides a mature template for standardization, reference ranges, and reporting (including effect sizes and profile-type reasoning) (14). Translating those norms to penile VPT would improve reproducibility and comparability across centers, and enable longitudinal within-patient comparison, turning repeated VPT testing into a quantitative monitor for sensory rehabilitation after targeted therapy.

Clinically, VPT can be positioned where it is strongest: (i) baseline phenotyping—a ten-site mean and site-pattern profile provide a compact descriptor of sensory thresholds; (ii) response tracking—longitudinal change after targeted management (e.g., prostatitis therapy or spine interventions) can be quantified if minimal detectable change and test-retest metrics are reported; and (iii) triage to functional studies when thresholds and symptoms are discordant. For the latter, pudendal SEPs (and related measures) remain the appropriate objective tests to interrogate pathway integrity; contemporary work continues to refine SEP methodology and shows its clinical utility in sexual neurophysiology (15). Early studies even suggest SEP differences across PE subtypes (16), underscoring how structural/threshold phenotypes could be paired with functional readouts in future prospective designs. From a practical standpoint, markedly elevated VPT with AE-type symptomatology should prompt lumbosacral imaging to rule out segmental conduction disturbance, whereas normal VPT with refractory PE symptoms may justify cervical screening to assess descending facilitatory circuits. In routine follow-up, repeating VPT after intervention provides an internal control for sensory recovery, anchoring subjective improvement to measurable change.

The differences in spinal imaging between AE and PE provide a distributional signal that is physiologically plausible within current models of ejaculatory control. Ejaculation is organized through a lumbosacral spinal generator modulated by descending inputs, and cervical involvement has been implicated in modifying sensory integration and autonomic outflow (17, 18). Descending projections from the paraventricular nucleus and medial preoptic area travel through the dorsolateral and ventrolateral funiculi to reach the intermediolateral cell column (IML) and Onuf's nucleus, where they coordinate sympathetic emission and somatic expulsion. Disruption of these tracts at the cervical level, even if subclinical, can desynchronize descending inhibition and lead to premature activation of sacral reflex loops, offering a mechanistic substrate for PE. In our study, PE was disproportionately associated with isolated cervical abnormalities, whereas AE more often displayed either normal imaging or combined cervical–lumbar changes—suggesting that descending modulation may dominate in PE, while AE reflects multi-segmental or afferent disruption (19). Cervical spondylotic or ischemic changes may impair conduction in long descending tracts, whereas AE's combined or lumbar-predominant lesions likely involve the dorsal horn interneuronal circuits and pudendal afferents entering at S2–S4, attenuating the sensory feedback that normally amplifies the emission phase. This interruption weakens excitatory drive to IML sympathetic neurons and Onuf's motoneurons, resulting in failure to reach the excitatory threshold for coordinated emission and expulsion.

While many of these spinal alterations are degenerative rather than inflammatory, their segmental distribution aligns with the functional architecture of the ejaculation reflex arc—cervical lesions influencing descending timing control, lumbar and sacral lesions disturbing the local reflex integration. Such anatomical correspondence reinforces the likelihood that these MRI findings, while often subtle, are functionally relevant rather than coincidental. Age-related spondylosis and metabolic comorbidities such as diabetes could further impair cord microcirculation and axonal myelination, amplifying these effects over time. Methodologically, our use of four mutually exclusive categories—no abnormality, cervical only, lumbar only, and combined cervical–lumbar changes—was designed to capture the topographic pattern of involvement rather than to dichotomize findings into “normal” or “abnormal” (20). This categorical approach offers finer granularity than prior descriptive reports but remains inherently observational, emphasizing spatial distribution over causality. Clinically, the relevance lies not in assigning diagnostic weight to an individual MRI but in focusing attention: in PE, cervical imaging deserves scrutiny, while in AE, the presence of normal scans or dual-segment findings should not be dismissed as inconsistent with clinical presentation. The boundary is clear—these are population-level tendencies, not mechanistic diagnoses, and imaging should be interpreted in conjunction with symptoms and examination rather than in isolation.

The markedly higher prevalence of chronic prostatitis in AE than PE also merits careful positioning. Mechanistically, chronic prostatic inflammation can alter seminal tract physiology, sensory input, and pelvic pain pathways, offering a plausible link to ejaculatory dysfunction, but cross-sectional association cannot separate cause from comorbidity (21). Methodologically, we relied on established diagnostic criteria—microscopy of expressed prostatic secretions supported by characteristic ultrasound features—thereby minimizing misclassification (22, 23). The clinical message is more straightforward: in AE pathways, systematic assessment of prostatitis is warranted, both for symptom management and for complete documentation; in PE, where prevalence is substantially lower, reflex attribution of ejaculatory symptoms to prostatitis should be avoided. The boundary again is critical—our findings identify differential burden, not causal attribution, and the essential next step is prospective testing of whether targeted prostatitis treatment produces measurable within-patient improvements in ejaculatory outcomes or in sensory thresholds.

The psychological assessments add a further layer to understanding how AE and PE differ, though the signal was narrower than commonly assumed. Mechanistically, depressive symptoms can influence sexual motivation, arousal, and central processing of sensory input, potentially contributing to impaired ejaculatory function. Functional neuroimaging and neuroendocrine data converge on this interpretation: reduced dopaminergic signaling within the mesolimbic pathway (ventral tegmental area–nucleus accumbens–medial prefrontal cortex) and hypoactivity of the hypothalamic–pituitary–gonadal axis in depression collectively blunt reward anticipation and autonomic arousal. These neurobiological changes compromise the initiation phase of ejaculation, which relies on coordinated activation of the paraventricular nucleus (PVN), periaqueductal gray, and lumbosacral spinal generator. The modest yet consistent elevation of PHQ-9 scores in AE therefore likely reflects a genuine neurofunctional inhibition of sexual drive and emission readiness, rather than a coincidental comorbidity (24).

In contrast, generalized anxiety and sexual desire, while long speculated to play roles, did not separate the groups in our cohort, suggesting that not all psychological domains exert equal impact. Anxiety primarily amplifies autonomic vigilance via the amygdala and locus coeruleus systems, producing transient sympathetic surges that may not directly engage the spinal emission circuitry; thus, generalized anxiety may heighten discomfort without altering the motor output of ejaculation. Similarly, sexual desire inventories assess motivational readiness but not sensory gating or reflex excitability. The absence of group differences could therefore stem from both physiological dissociation and the limited granularity of tools such as GAD-7 and SDI-2. More specialized instruments—sexual distress indices, relational satisfaction scales, or fMRI-based affective connectivity measures—could better delineate the affective circuits that modulate ejaculatory timing.

Methodologically, we used validated Chinese-language versions of PHQ-9, GAD-7, and SDI-2 administered by trained staff, ensuring standardized measurement and reducing cultural bias in scale interpretation (25). Nonetheless, these scales quantify symptom severity rather than dynamic affective processing. Longitudinal integration of psychometric, endocrine, and neurofunctional data will be crucial to determine whether alleviating depressive symptoms—pharmacologically or behaviorally—translates into improved ejaculatory performance. Clinically, our findings suggest that systematic mood screening should become a standard part of andrological evaluation: AE patients with elevated PHQ-9 scores may benefit from concurrent psychiatric or neuroendocrine consultation, whereas anxiety-focused assessment may be reserved for broader psychosocial profiling. Embedding these evaluations into routine workflows could enhance early detection of mood-linked ejaculatory dysfunction and support tailored therapeutic strategies. The boundary remains that self-report scales measure perceived burden, not causation; prospective studies combining mood trajectory, neuroimaging, and treatment response are needed to establish mechanistic directionality.

A few pragmatic constraints merit note. Stopwatch-verified intravaginal ejaculation latency time (IELT) and standardized erectile function indices were not part of the routine clinic workflow (26), so analyses centered on validated symptom scales and objective sensory/imaging measures. Precision for very small subgroups (e.g., cervical-only findings within AE) is limited; we therefore reported exact *p* values, interpreted such cells cautiously, and emphasized cohort-level patterns. Within-visit repeatability of VPT was not separately tested; to stabilize variability we used a standardized ten-site protocol and summarized thresholds with both a composite mean and a multivariate profile (27). These considerations are unlikely to alter the direction of the findings, but, given the retrospective cohort design, causal inference and the temporal ordering among sensory, spinal, and affective changes cannot be established; the lack of longitudinal follow-up likewise prevents assessing whether these phenotypic signatures remain stable or respond to treatment over time. They delineate clear priorities for prospective work. Future studies should extend these findings through prospective, multicenter designs that combine quantitative spinal metrics, functional neurophysiology, and longitudinal psychological assessment, ideally incorporating standardized latency and erectile-function metrics to enhance comparability with latency-indexed literature—with the goal of establishing whether modifiable factors can predict treatment response and improve patient outcomes.

## 5 Conclusion

Anejaculation (AE) and premature ejaculation (PE) differ not only in clinical presentation but also in their underlying profiles. AE was associated with elevated vibration perception thresholds, higher prevalence of chronic prostatitis, and greater depressive symptom burden, whereas PE more frequently showed isolated cervical spine abnormalities. These distinctions highlight divergent pathophysiological processes and underscore the need for tailored evaluation in clinical practice.

## Data Availability

The original contributions presented in the study are included in the article/Supplementary Material, further inquiries can be directed to the corresponding author.
